# NfL concentration in CSF is a quantitative marker of the rate of neurodegeneration in aging and Huntington's disease: a semi-mechanistic model-based analysis

**DOI:** 10.3389/fnins.2024.1420198

**Published:** 2024-07-03

**Authors:** Matthias Machacek, Elena Garcia-Montoya, Peter McColgan, Patricia Sanwald-Ducray, Norman Alan Mazer

**Affiliations:** ^1^LYO-X AG, Basel, Switzerland; ^2^Roche Products Limited, Welwyn Garden City, United Kingdom; ^3^Roche Pharmaceutical Research and Early Development, Roche Innovation Center Basel, F. Hoffmann-La Roche Ltd, Basel, Switzerland; ^4^NAM Consulting, Pfeffingen, Switzerland

**Keywords:** neurodegeneration, atrophy, Huntington's disease, neurofilament light chain, cerebrospinal fluid, mathematical, model, biomarker

## Abstract

The concentrations of neurofilament light chain (NfL) in cerebrospinal fluid (CSF) and plasma have become key biomarkers of many neurodegenerative diseases, including Huntington's Disease (HD). However, the relationship between the dynamics of NfL concentrations in CSF and the time-course of neurodegeneration (whole brain atrophy) has not yet been described in a quantitative and mechanistic manner. Here, we present a novel semi-mechanistic model, which postulates that the amount of NfL entering the CSF corresponds to the amount of NfL released from damaged neurons, whose degeneration results in a decrease in brain volume. In mathematical terms, the model expresses the NfL concentration in CSF in terms of the NfL concentration in brain tissue, the rate of change of whole brain volume and the CSF flow rate. To test our model, we used a non-linear mixed effects approach to analyze NfL and brain volume data from the HD-CSF study, a 24-month prospective study of individuals with premanifest HD, manifest HD and healthy controls. The time-course of whole brain volume, obtained from MRI, was represented empirically by a 2nd order polynomial, from which its rate of change was computed. CSF flow rates in healthy and HD populations were taken from recent literature data. By estimating the NfL concentration in brain tissue, the model successfully described the time-course of the NfL concentration in CSF in both HD subjects and healthy controls. Furthermore, the model-derived estimate of NfL concentration in brain agreed well with recent direct experimental measurements. The consistency of our model with the NfL and brain volume data suggests that the NfL concentration in CSF reflects the rate, rather than the extent, of neurodegeneration and that the increase in NfL concentration over time is a measure of the accelerating rate of neurodegeneration associated with aging and HD. For HD subjects, the degree of acceleration was found to increase markedly with the number of CAG repeats on their HTT gene. The application of our semi-mechanistic NfL model to other neurodegenerative diseases is discussed.

## 1 Introduction

Neurofilament light chain (NfL), a 61.5 kDa structural protein exclusive to neurons, combines with medium and heavy chain (NfM and NfH) neurofilaments to form type IV intermediate fibers (Khalil et al., 2018). Upon axonal damage or neuronal death, NfL is released to the extracellular space where it is removed via microglia- and CSF-mediated mechanisms (Khalil et al., 2018; Kölliker Frers et al., 2022). The concentrations of NfL in cerebrospinal fluid (CSF) and plasma are purported markers of neurodegeneration in a number of neurological diseases (Khalil et al., 2018; Gaetani et al., 2019). In Huntington's disease (HD), for example, elevated NfL levels are a highly sensitive marker of disease onset and progression (Rodrigues et al., 2020; Scahill et al., 2020). The elevations of NfL are associated in HD with the number of CAG repeats found on the mutant HTT gene (Rodrigues et al., 2020).

Despite the relevance of NfL as a biomarker of neurodegeneration, the mechanistic link between neuronal damage and NfL concentration in CSF has not been well described (Gafson et al., 2020). In particular, the quantitative relationships between NfL concentrations in biofluids such as CSF, blood, serum or plasma and the extent or rate of neurodegeneration in various disease states is still not well defined. In the case of the blood compartment, into which NfL enters from the CSF, a number of physiological and demographic variables have been shown to influence the NfL concentration. These include blood volume and BMI, which are inversely related to NfL levels (Manouchehrinia et al., 2020); diabetes, COPD, cardiovascular disease, and COVID-19 infection, which are associated with increased NfL levels (Fitzgerald et al., 2022; Abdelhak et al., 2023); and pre-eclampsia and parturition, in which NfL levels are also elevated (Evers et al., 2018, 2019). In contrast, the NfL concentrations in CSF are appreciably higher than in blood and provide a more direct assessment of NfL input from the brain. They are primarily influenced by CSF flow rates, rather than the confounders that affect NfL in the blood compartment (Andersson et al., 2020; Manouchehrinia et al., 2020; Tang et al., 2022).

The usefulness of NfL as a biomarker of neurodegeneration is, presumably, due to its neuron-specific location, high relative expression, cellular release during axonal damage or degeneration, and size that enables CSF-mediated clearance. Furthermore, the inability of central nervous system neurons to proliferate or regenerate in adulthood (Huebner and Strittmatter, 2009; Sorrells et al., 2018) suggests that NfL release is not a consequence of turnover and instead results from neuronal damage or death.

Here, we characterize the quantitative relationships between MRI measurements of whole brain volume, NfL concentration in CSF, and measurements of CSF flow rates in healthy controls and HD subjects using published data from the HD-CSF study (Rodrigues et al., 2020) and a separate study of CSF flow (Hett et al., 2023). Based on mass-balance principles, we derive a novel, semi-mechanistic model that relates the NfL concentration in CSF to the rate of change of whole brain volume, the respective CSF flow rates, and the estimated NfL concentration in brain tissue. An empirically chosen 2nd order polynomial is used to represent the time-course of whole brain volume in healthy controls and HD subjects, from which its rate of change is computed. We use a population non-linear mixed effects (NLME) approach to estimate population parameters across all data sources combined.

Our semi-mechanistic model is shown to successfully predict the longitudinal and age-related changes in whole brain volume and NfL concentration in CSF in both healthy controls and HD populations over the age range of 26–77 years. Thus, in both cases, we conclude that the amount of NfL that is eliminated via CSF over a certain time period equals the amount of NfL contained in the volume of brain that is lost over the same time period. In essence, this means that the NfL concentration in CSF reflects the rate, rather than the extent, of neurodegeneration. Moreover, the increase in NfL concentration over time is a measure of the accelerating rate of neurodegeneration associated with aging and HD. In addition, our model provides an estimate of the average concentration of NfL in brain tissue that can be compared to direct biochemical measurements in post-mortem subjects (Sjölin et al., 2022) as a validation test. As a further test, the model is used to simulate an independent cross-sectional study of brain mass vs. age in healthy subjects (Svennerholm et al., 1997).

We conclude by discussing the application of our model of NfL to other neurodegenerative conditions along with its mechanistic and clinical implications.

## 2 Methods

### 2.1 Data acquisition

Individual data from the HD-CSF study (Rodrigues et al., 2020) for healthy controls (*n* = 20; mean age 50.7 yr), premanifest HD subjects (*n* = 20; mean age = 42.4 yr; mean CAG repeats = 42.0) and manifest HD subjects (*n* = 40; mean age = 56.0 yr; mean CAG repeats = 42.75) were downloaded from the UCL website (University College London, 2020). The following variables were used in the present analysis: ID, sample_day, gender, CAG (for premanifest and manifest HD subjects), age, group, wb_adj (adjusted whole brain volume, mL), and NfL_CSF2 (NfL concentration in CSF measured by assay 2; 4-Plex B, Quanterix, pg/mL).

Details of the MRI methodology used for measuring whole brain volume in the HD-CSF study can be found in prior publications (Byrne et al., 2018; Rodrigues et al., 2020) and are briefly summarized here. T1-weighted MRI data were acquired on a single 3T Siemens Prisma scanner. Images were acquired using a 3D magnetization-prepared 180 degrees radio-frequency pulses and rapid gradient-echo (MPRAGE) sequence with a repetition time (TR) = 2,000 ms and echo time (TE) = 2.05 ms. The acquisition had an inversion time of 850 ms, flip angle of 8 degrees, matrix size 256 × 240 mm. 256 coronal partitions were collected to cover the entire brain with a slice thickness of 1.0 mm. A semi-automated segmentation procedure was performed via Medical Image Display Analysis Software (MIDAS) to generate volumetric regions of the whole-brain and Total Intracranial Volume (TIV). Follow-up whole-brain volume was measured via the same semi-automated procedure. The adjusted total brain volume was computed as the ratio of whole brain volume-to-TIV, multiplied by the mean TIV of the population (personal communication from R. Scahill). The median net CSF flow rates (μL/min) for healthy controls (*n* = 51), premanifest HD (*n* = 17) and manifest HD (*n* = 12) measured by MRI methods were obtained from a separate study (Hett et al., 2023).

For the validation testing of the model, individual post-mortem brain mass data were obtained from the Svennerholm study (Svennerholm et al., 1997) in females (*n* = 83) and males (*n* = 101) ranging in age from 20 to 100 years, with no prior history or pathological evidence of neurological disease. Svennerholm's data were digitized using the DigitizeIt software (Bormann, 2016). To convert mass (g) to volume (mL), the density of brain tissue was taken to be 1.03 g/mL (Berger et al., 2017).

### 2.2 Modeling

The assumptions and mathematical derivation of the semi-mechanistic model describing the dynamics of the NfL concentration in CSF and its relationship to the time-course of whole brain volume are given in Results (Section 3.1). The equations used to model the time-course of whole brain volume in the healthy controls and HD subjects are given in Results (Section 3.2). Values of the CSF flow rates for healthy controls and HD subjects, which are equivalent to the NfL clearances rates in CSF, were obtained from literature data, as discussed in Results (Section 3.3). The remaining model parameters were estimated from an integrated analysis of the brain volume and NfL concentration in CSF reported in the HD-CSF study using a non-linear mixed effects analysis in Monolix 2023R1. Details of the parameter estimation, statistical model, covariate analysis and model-based simulations are given in the Supplementary material (Section 1).

### 2.3 Software and computer systems

All data programming, data exploration, model building, and simulations were done on a desktop computer (PowerCrunch-8) running Windows 10 Professional. For parameter estimation and diagnostic plots, a validated version of Monolix 2023R1 was used (Monolix 2023R1). For simulations, a validated version of Simulx 2023R1, and a validated version of R 4.3.2 (R Core Team, 2021) were used.

## 3 Results

### 3.1 Development of a semi-mechanistic model of NfL dynamics in CSF

In developing our semi-mechanistic model of NfL dynamics in CSF, we made the following simplifying assumptions:

1. NfL is only contained in neurons, which cannot regenerate or turn over.
2. Decreases in whole brain volume reflect loss of neurons, glial cells and interstitial fluid.
3. NfL concentration in brain tissue is the same for healthy controls and HD subjects and is assumed to be homogenous throughout the brain.
4. NfL is released from neurons only upon axonal damage or cell death.
5. All NfL molecules released (including any proteolytic fragments) enter the CSF and are cleared from the brain by bulk CSF flow.
6. CSF flow rates may differ between healthy controls and HD subjects and can potentially vary with age.

**Figure 1:**
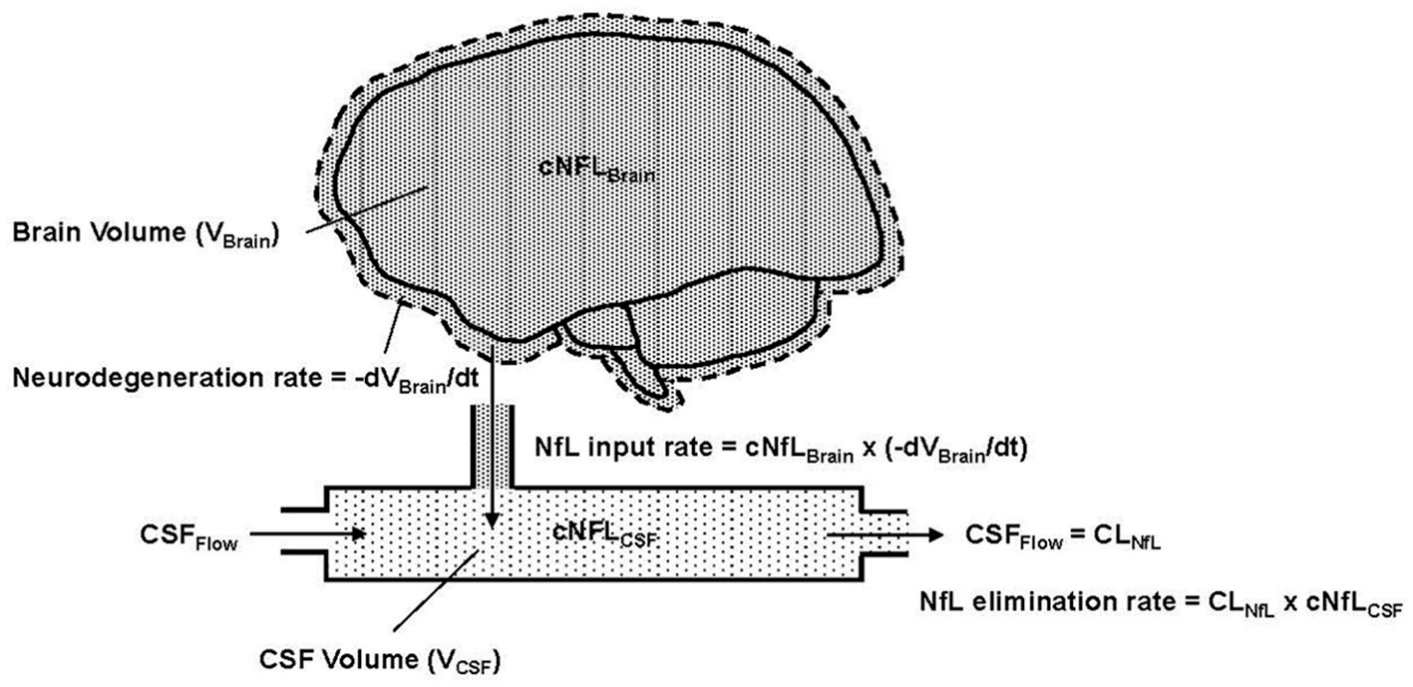
Conceptual representation of NfL in the brain and CSF, where cNFL_Brain_ (dense pattern) and cNFL_CSF_ (light pattern) indicate the concentrations of NfL in brain tissue and CSF, respectively. V_Brain_ is the volume of brain tissue, V_CSF_ is the volume in the CSF compartment and t is the time variable, which is also equivalent to age in the model. –dV_Brain_/dt is the negative rate of change of the brain volume; the negative sign expresses this as a positive rate of neurodegeneration. Flow into and out of the CSF compartment is denoted CSF_Flow_. The observed NfL concentration in CSF can change due to alterations in the rate of neurodegeneration (NfL input to CSF) or CSF_Flow_ (NfL elimination from CSF). The latter is physiologically equivalent to CSF clearance, denoted CL_NfL_.

Combining these mechanistic assumptions with the principles of mass balance, we illustrate in Figure 1 our conceptual model of NfL dynamics in the brain and CSF. Qualitatively, the figure shows that the loss of brain volume releases NfL into the CSF (represented as a single compartment). The NfL in CSF is then cleared by CSF flow.

Based on assumptions 1–4, Equation 1 states that the NfL input rate into the CSF (mass/time) is the product of the NfL concentration in brain tissue (denoted cNfL_Brain_) and the neurodegeneration rate, expressed as the negative rate of change of brain volume (-dV_Brain_/dt).


(1) $$NfL\;input\;rate\;=\;cNfL_{Brain}\cdot \left(-\frac{dV_{Brain}}{dt}\right),\;\frac{dV_{Brain}}{dt}<0,$$

Based on assumption 5, the elimination rate of NfL from the CSF compartment is equal to the product of NfL concentration in the CSF (denoted cNFL_CSF_) and the CSF flow rate, which, in principle, corresponds to the clearance rate of NfL from the CSF (denoted CL_NfL_). Equation 2 expresses the rate of change of NfL concentration in the CSF compartment as the difference between the NfL input rate and NfL elimination rate, divided by the CSF volume (V_CSF_).


(2) $$\frac{dcNfL_{CSF}}{dt}\;=\frac{\left[cNfL_{Brain}\cdot \left(-\frac{dV_{Brain}}{dt}\right)-CL_{NfL}\left(t\right)\times cNfL_{CSF}\left(t\right)\right]}{V_{CSF}},$$

Due to the fast rate of CSF turnover (ca. hours) relative to the slow timescale of chronic neurodegeneration (ca. months to years), one can reasonably assume that the NfL concentration in CSF is in a quasi-steady-state (i.e., dcNfL_CSF_/dt) ≈ 0). Equation 2 then reduces to Equation 3, which states that the NfL concentration in CSF is equal to the NfL concentration in brain multiplied by the negative rate of change of brain volume, divided by the NfL clearance rate.


(3) $$\begin{array}{l} cNfL_{CSF}\left(t\right)=\frac{cNfL_{Brain}\cdot \left(-\frac{dV_{Brain}}{dt}\right)}{CL_{NfL}\left(t\right)}, \end{array}$$

The ability of Equation 3 to predict the dynamics of cNfL_CSF_ seen in the HD-CSF study will be presented after describing how the time-course of whole brain volume was modeled and the CSF flow rates (CL_NfL_) were estimated in healthy controls and HD patients (consistent with assumption 6).

### 3.2 Modeling the time-course of whole brain volume

**Figure 2:**
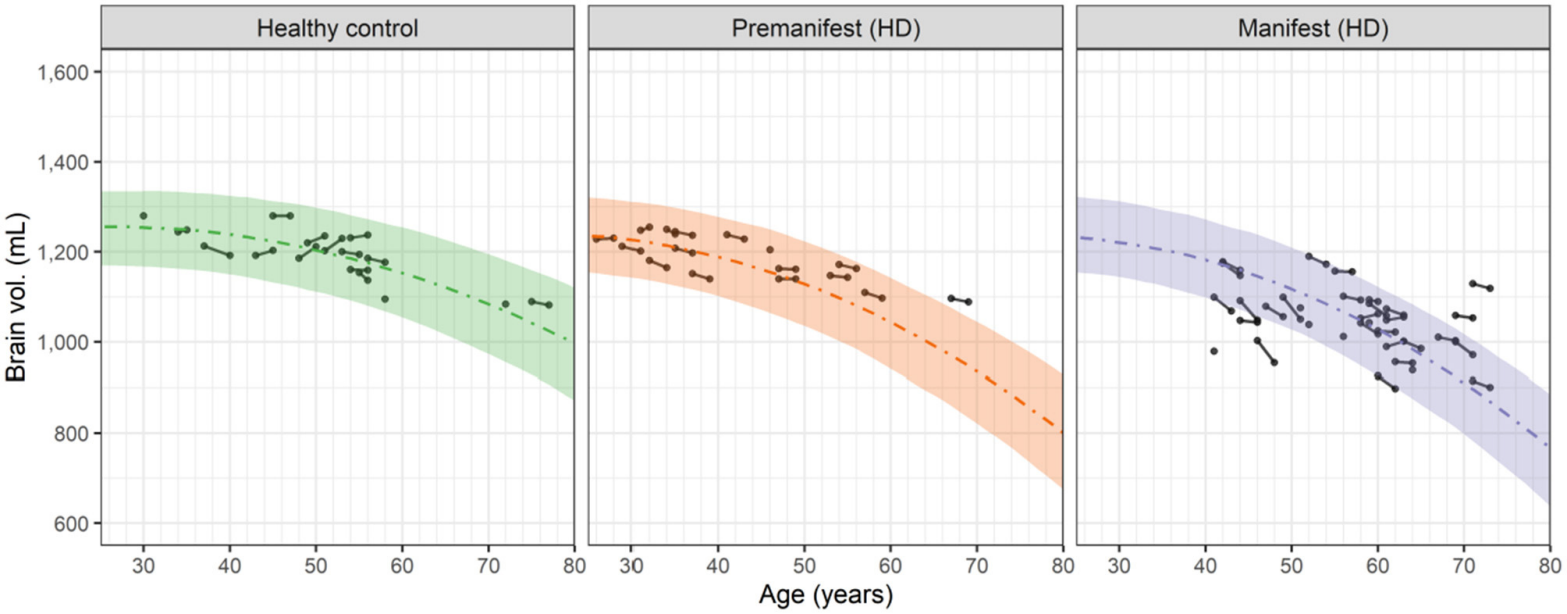
Observed and predicted dependence of the whole brain volume vs. age for healthy controls, premanifest HD subjects and manifest HD subjects in the HD-CSF study. Filled circles represent individual observations with line segments connecting the baseline and 24-month follow up measurements. A small number of data points had no follow up measurement. Dashed curves correspond to the predicted median values for each group based on the 2nd order polynomials in Equation 4. For premanifest and manifest HD subjects, k_2_ was computed from Equation 6 using the mean CAG repeat values of 42.0 and 42.75, respectively. Shaded areas represent the 90 percent prediction intervals of the model.

Visual inspection of the longitudinal and cross sectional relationship between the whole brain volume and subject's age (which increased by 2 years during the study) suggested that a 2nd order (quadratic) polynomial could empirically describe the observed data in the HD-CSF study (Figure 2).

Equation 4 expresses this quadratic dependence as:


(4) $$\begin{array}{l} V_{Brain}\left(t\right)=k_{0}+k_{1}t-k_{2}t^{2}, \end{array}$$

where *V*_*Brain*_(*t*) is the whole brain volume at time t (equivalent to age) and *k*_0_ (in mL), *k*_1_ (in mL/yr) and *k*_2_ (in mL/yr/yr) are the three polynomial coefficients. From this equation, the rate of neurodegeneration, i.e., negative rate of change of *V*_*Brain*_(*t*), is given by Equation 5.


(5) $$\begin{array}{l} -\;\frac{dV_{Brain}}{dt}\left(t\right)={-\;k}_{1}+{2\;k}_{2}t, \end{array}$$

This result shows that the neurodegeneration rate increases linearly in time (or age) from the basal rate of − *k*_1_ with a slope (acceleration) of 2 *k*_2_. Fitting Equation 4 to the brain volume data in Figure 2 (simultaneously with fitting the cNfL_CSF_ data, as described in Section 3.4) showed that the estimated value of *k*_1_, i.e., 4.84 mL/yr, was the same for healthy controls and HD subjects. In contrast, the value of *k*_2_ in healthy controls, i.e., 0.0902 mL/yr/yr, was smaller than the *k*_2_ value in HD subjects. The latter was found to increase markedly with the CAG repeat value based on a covariate analysis (see Supplementary material; Section 1). Equation 6 describes this relationship:


(6) $$\begin{array}{l} k_{2\;HD,\;i}=k_{2\;HD,\;Pop}\times \;(\frac{CAG_{i}}{42.5}){\;}^{\beta_{CAG}}, \end{array}$$

where *k*_2 *HD, i*_ is the *k*_2_ value for HD subject *i* with a CAG repeat value of *CAG*_*i*_, *k*_2 *HD, Pop*_ is the *k*_2_ value for the overall HD population with the mean CAG repeat value of 42.5, estimated to be 0.125 mL/yr/yr, and β_*CAG*_ is a dimensionless exponent, estimated to be 2.74.

**Figure 3:**
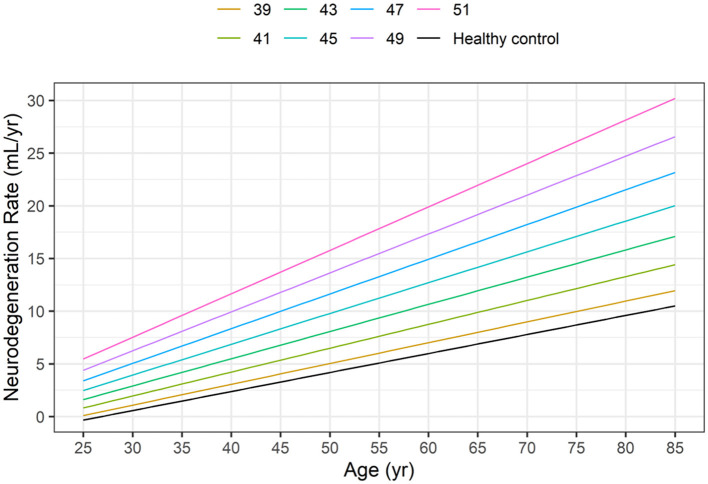
Dependence of neurodegeneration rate (–dV_Brain_/dt) on age in healthy controls (HC) and in HD subjects as a function of the CAG repeat length based on Equations 5, 6.

Based on Equations 5, 6 and Figure 3 illustrates the neurodegeneration rate as a function of age (time) for healthy controls and HD subjects with CAG values ranging from 39 to 51, as in the HD-CSF dataset.

At any given age, the model-based neurodegeneration rate in HD subjects increases markedly from the corresponding value in healthy controls, as the CAG repeat length increases from 39 to 51.

### 3.3 CSF flow rates in healthy controls and HD subjects

As depicted in Figure 1, it is assumed that the clearance rate of NfL from the CSF compartment is equal to the CSF flow rate. CSF flow rates (or equivalently, CSF production rates) have been reported for humans in health and disease using a variety of methods (Liu et al., 2022). According to current reviews (Khasawneh et al., 2018; Liu et al., 2022; Czarniak et al., 2023), the CSF production rate in healthy adults typically ranges between 300 and 400 μL/min, consistent with the early measurement of 350 μL/min by Davson et al. (1987) and the recent model-derived value of 380 ± 20 μL/min (Elbert et al., 2022).

The current gold standard for quantifying CSF production is regarded to be the MRI-based measurement of net CSF flow rate through the cerebral aqueduct (Liu et al., 2022). Using this technique, Hett et al. (2023) recently reported the median values of the net CSF flow rate to be 303 μL/min in healthy controls (mean age 46.6 yr; CAG < 37), 136 μL/min in premanifest HD subjects (mean age 41.5 yr; mean CAG 42) and 108 μL/min in manifest HD subjects (mean age 45.9 yr; mean CAG 45). Given the consistency of the healthy control value with the normative data summarized above, we have taken the three median values from Hett's study as fixed parameter estimates of the CL_NfL_ values for the respective groups in our model. Based on the findings of Elbert's study, we assume that in healthy subjects, CL_NfL_ is not dependent on age (or time).

### 3.4 Neurodegeneration rates and CL_*NfL*_ values predict CSF NfL

Having shown in Figure 2 that Equations 4–6 of our model can successfully describe the time-course of brain volume in healthy controls and HD subjects, we assess here the ability of Equation 3 to predict the time-course of the NfL concentrations in CSF observed in the HD-CSF study. To do this, the individual rates of neurodegeneration derived from Equations 5, 6 were combined with the median values of CL_NfL_ from Hett's study, and the value of the NfL brain concentration (cNfL_Brain_) was estimated by fitting the model to the cNfL_CSF_ data. Details of the mixed effects modeling approach used for simultaneously estimating all model parameters and their population variances are given in the Supplementary material (Section 1).

**Figure 4:**
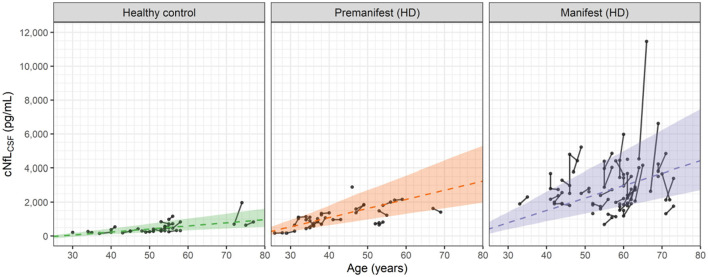
Observed and predicted dependence of the NfL concentrations in CSF (cNfL_CSF_) vs. age for healthy controls, premanifest HD subjects and manifest HD subjects in the HD-CSF study. Filled circles represent individual observations with line segments connecting the baseline and 24-month follow up measurements. A small number of data points had no follow up measurement. Dashed lines correspond to the predicted median values for each group based on Equations 3, 5, 6. For premanifest and manifest HD subjects, k_2_ was computed from Equation 6 using the mean CAG repeat values of 42 and 42.75, respectively. Shaded areas represent the 90 percent prediction intervals of the model.

Figure 4 shows the longitudinal and cross-sectional dependence of the cNfL_CSF_ data on age for the healthy controls, premanifest HD subjects and manifest HD subjects.

In general, the linear cross-sectional trends in all three groups are well predicted by the age-dependences of the model. The longitudinal changes for the healthy controls and premanifest HD subjects are also well predicted. In the manifest HD subjects, however, a number of individuals exhibit sharp increases that exceed the 90% prediction interval of the model.

We attribute these sporadic increases to observational or measurement errors in the cNfL_CSF_ values, which the model also estimates when fitting the data. In Section 2 of the Supplementary material, graphical assessments of the observational errors in both the NfL concentrations and brain volume data are given. For cNfL_CSF_, the observational error is proportional to the measured value with a coefficient of 0.216 (equivalent to a coefficient of variation of ca. 22%). For large values of cNfL_CSF_, e.g., 5,000 pg/mL, the observational error can be sizeable, with a standard deviation of ca. 1,000 pg/mL. In constrast, for the brain volume data the observational error is found to be additive, with a standard deviation of 11 mL, independent of the measured value. Observational errors can therefore account for the small increases in brain volume seen occasionally in Figure 2. As shown in the Supplementary material (Section 2) the standard goodness of fit measures used to assess non-linear mixed effect models of this type demonstrate that the model provides a robust description of the cNfL_CSF_ and brain volume data, as well as the observational errors associated with them.

Additional analysis of the model, exploring the effect of the CAG repeat length on the age-dependences of V_Brain_ and cNfL_CSF_, is given in Supplementary material (Section 3) and further supports the robustness of the model.

### 3.5 Model derived estimate of the NfL concentration in brain tissue

As an outcome of modeling the NfL concentrations in CSF by Equation 3, we obtained an estimate of the NfL concentration in brain tissue (cNfL_Brain_) equal to 15.5 μg/g (assuming a tissue density of 1.03 g/mL; Berger et al., 2017). This value is in good agreement with the range of experimental NfL concentrations, 10 to 47 μg/g, measured by ELISA in post-mortem brain and spinal cord tissue samples from 10 neurologically healthy subjects (Sjölin et al., 2022).

## 4 Discussion

We have developed a novel quantitative, semi-mechanistic model of the dynamics of NfL concentrations in CSF and its relationship to neurodegeneration, based on an analysis of data from healthy controls and HD subjects in the HD-CSF study (Rodrigues et al., 2020). Our principal findings are that the NfL concentration in CSF is directly proportional to the rate, rather than the extent, of neurodegeneration (defined as the negative rate of change of whole brain volume) and is inversely proportional to the flow rate of CSF, which is reduced in HD subjects relative to healthy subjects (Hett et al., 2023). The linear increase of the NfL concentration in CSF with age (or time), observed in both healthy controls and HD subjects, is explained in our model by the acceleration of the neurodegeneration rate, which is quantified by modeling the time-course of brain volume with a 2nd order polynomial, i.e., a quadratic dependence. The degree of acceleration in HD subjects is further shown to increase strongly with their CAG repeat length. Our findings agree with, and put into mechanistic context, previous findings on the relationship between the rate of brain atrophy and NfL levels in blood serum (Khalil et al., 2020, 2024; Gallingani et al., 2024).

It may be noted that we also tested a linear model for the age/time dependence of the brain volume data and found that it was incompatible with the increase in NfL concentrations observed in all three groups of subjects. The corrected Bayesian Information Criterion, a statistical measure of the goodness of fit, confirmed the overall superiority of the quadratic dependence (see Supplementary material, Section 1.1). Additional support for the 2nd order polynomial used in our model is given in the Supplementary material (Section 4), where it is shown that the same function describes well the cross-sectional relationship between brain mass and age in a different population of neurologically healthy subjects studied post-mortem by Svennerholm et al. (1997). Whitwell also used a quadratic function to represent the longitudinal trajectory of brain volume data in an AD patient studied over 6.8 years (Whitwell et al., 2001).

We believe that the strengths of our model are its simplicity, use of physiologic mechanisms, ability to describe brain volume and NfL data from healthy controls and HD subjects, and the incorporation of CSF flow measurements reported in populations that are similar to those in the HD-CSF study. Our model differs in a number of important ways from the mathematical model developed by Paris et al. (2022) to describe the age-dependence of NfL (and other neurofilament species) in CSF and plasma from healthy subjects. While both models are based on compartmental analysis and physiologic principals, the Paris model has more than 13 parameters and does not incorporate any changes in brain volume. It assumes that in healthy subjects the leakage rate of NfL from neurons is not caused by neurodegeneration and instead, uses an empirical function to modulate the leakage rate constants in proportion to age. In contrast, the present model accounts for the age-related increase in NfL concentrations seen in healthy controls and HD subjects by the acceleration of the neurodegeneration rate inferred from the brain volume data. Recently, the Paris model was adapted to analyze the dynamics of neurofilament heavy chain (NfH) in pediatric patients with spinal muscular atrophy (SMA) and the effects of treatment (Paris et al., 2023). The adapted SMA model included an extra term to characterize the increased leakage rate constant of NfH from the spinal neuron compartment and its downregulation during treatment.

Further support for the model derives from our estimate of the NfL concentration in brain tissue, which is found to be in good agreement with the experimental data reported in post-mortem samples of brain and spinal cord using ELISA methods that are similar to those used in the HD-CSF study (Sjölin et al., 2022). Such agreement provides important validation of the mass-balance principles used in the model and suggests that most of the NfL released from damaged neurons in the brain appears in the CSF. An early study of neurofilament levels in human brain tissue using Western blot methods obtained NfL concentrations that were approximately 10-fold higher than the ELISA results when converted to μg/g (Ferrer-Alcón et al., 2000). We have found no other experimental data in the literature that can be compared to our model-based estimate of cNfl_Brain_. In this regard, the detailed immunoprecipitation-mass spectrometry study of NfL molecular species by Budelier showed that in brain tissue, NfL is present mostly as a full-length, intact molecule, while in CSF it is present in at least three main fragments (Budelier et al., 2022). Unfortunately, Budelier's characterization of NfL in brain tissue is not readily expressible in the μg/g units needed for comparison with our estimate of cNfL_Brain_. While recent literature suggests that NfL fragments may be secreted in exosomes (Zanardini et al., 2022), it is unclear to what extent exosomal NfL derived from healthy neurons may contribute to the concentrations in biofluids. In the context of our model, we believe that exosomal NfL would represent a relatively small and roughly constant addition to the CSF NfL concentration and not materially alter the results.

Limitations of our model primarily reflect the relatively small number of HD subjects in the HD-CSF and Hett studies. Concerns have also been raised about the MRI methods for quantifying net CSF flow (Liu et al., 2022), including diurnal variation (Nilsson et al., 1992) and the effects of respiration (Spijkerman et al., 2019). In this regard, any systematic errors in the assumed values of CL_CSF_ will be compensated for in our model by the estimated value of cNfL_Brain_, thereby preserving the agreement between the predictions of Equation 3 and the cNfL_CSF_ data. Lastly, the model is currently limited to describing NfL dynamics in CSF rather than plasma where similar changes with age and neurodegenerative disease have been observed (Khalil et al., 2018). For the reasons noted in the Introduction, extending the current model to the blood compartment will require a better understanding of the input, metabolism and clearance processes affecting circulating NfL in order to account for the observed effects of blood volume, BMI, renal function and other factors.

In principle, our semi-mechanistic model could apply to other chronic neurodegenerative conditions such as Amyotrophic Lateral Sclerosis, Alzheimer's disease, Multiple Sclerosis, Parkinson's disease and others (Khalil et al., 2018). In these other conditions, the validity of our model will depend on the mechanisms that release and clear NfL from damaged neurons. While for aging and HD, the data are consistent with the hypothesis that all of the NfL released from damaged neurons is cleared by CSF flow, it is possible that in other diseases some of the NfL released could be cleared prior to reaching the CSF. For example, in Alzheimer's disease it was reported that low plasma levels of NfL were associated with increased activation of cortical microglia (Parbo et al., 2020), suggesting in this case that NfL clearance by microglia could be an important contributor to the NfL mass balance. It is also conceivable that in some neurological conditions, NfL could be released into CSF from damaged neurons that might not fully degenerate and lead to measurable loss of brain volume. Such cases would require adaptions to Equation 1. Extending the application of our model to other diseases will require quantitative data on the time-course of brain volume, NfL concentrations in CSF, and ideally measurements of CSF flow in these states. In the case of modeling acute traumatic conditions where NfL increases sharply (Khalil et al., 2018), the quasi-steady-state assumption of Equation 3 may not be appropriate. In that case, Equation 2 will be required and the volume of the CSF compartment will have to be estimated or taken from the literature.

Finally, from the clinical perspective our model has two important implications. First, it suggests that the NfL concentration in CSF may indicate the current rate of neurodegeneration as opposed to the extent of neurodegeneration. Second, to assess the extent of neurodegeneration, the area under the NfL concentration-time curve over a relevant time interval would yield a measure proportional to the total brain volume lost during that time—a prediction of the model that is testable empirically. We believe these two points will benefit researchers investigating NfL in the context of neurodegeneration.

## Data availability statement

Publicly available datasets were analyzed in this study. This data can be found here: https://rdr.ucl.ac.uk/articles/dataset/HD-CSF_study_mhtt_and_NfL_dataset/12709391.

## Ethics statement

The HD-CSF study was approved by the London Camberwell St. Giles Research Ethics Committee (15/LO/1917). The Hett et al. (2023) study was approved by an unnamed local institutional review board. The Sjölin et al. (2022) study analyzed post-mortem brain samples obtained from the Karolinska Institute's tissue donation program. The studies were conducted in accordance with the local legislation and institutional requirements. Written informed consent for participation was not required from the participants or the participants' legal guardians/next of kin in accordance with the national legislation and institutional requirements.

## Author contributions

MM: Conceptualization, Writing – review & editing, Funding acquisition, Investigation, Methodology, Project administration, Supervision. EG-M: Data curation, Formal analysis, Investigation, Methodology, Software, Writing – review & editing. PM: Writing – review & editing. PS-D: Conceptualization, Funding acquisition, Investigation, Project administration, Supervision, Writing – review & editing. NM: Conceptualization, Investigation, Methodology, Validation, Visualization, Writing – original draft, Writing – review & editing.
