# Supplementary material for: NfL concentration in CSF is a quantitative marker of the rate of neurodegeneration in aging and Huntington's disease: a semi-mechanistic model-based analysis
Source: Front Neurosci. 2024 Jul 3;18:1420198. doi: 10.3389/fnins.2024.1420198 (PMC11253127; doi:10.3389/fnins.2024.1420198)
Supplement: Supplementary file 1 [file Data_Sheet_1.pdf]

Supplementary Material

NfL concentration in CSF is a quantitative marker of the rate of neurodegeneration in aging and Huntington's Disease: a semi-mechanistic model-based analysis

Matthias Machacek, Elena Garcia Montoya, Peter McColgan, Patricia Sanwald-Ducray* and Norman Alan Mazer*

*** Correspondence:** Corresponding Authors: patricia.sandwald_ducray@roche.com and namazer.consulting@gmail.com

1 Supplementary File 1: Non-linear mixed effect modeling of brain volumes and NfL concentrations in CSF

1.1 Parameter estimation

Population parameters were estimated using the stochastic approximation of expectation-maximization (SAEM) algorithm implemented in Monolix [Monolix 2023R1]. The maximum and minimum number of iteration steps for the exploratory search phase were set to 500 and 150, respectively. For the smoothing phase, the maximum and minimum steps were 200 and 50, respectively. It was confirmed with the SAEM convergence plots that the number of iteration steps was sufficiently large to have convergence to stable parameter estimates in all cases. The values for the NfL clearance in CSF (Cl_{NfL}) were fixed to the median net CSF flow rates for healthy controls, premanifest and manifest HD subjects reported by Hett [shown in Figure 2A of Hett 2022]. The standard errors of the parameter estimates were derived from the Fisher information matrix. For the individual parameters, the empirical Bayes estimates (EBEs), conditional mean and standard deviation were computed. EBEs were estimated using Nelder-Mead simplex algorithm with a minimum number of iterations of 200 and stopping tolerance of 0.000001. For the stopping rule of the individual parameter estimation, the Markov Chain Monte Carlo (MCMC) evaluation length was set to 50. Parameters with no variability were estimated using the "Add decreasing variability" feature of Monolix. The -2 log-likelihood (-2LL) was computed using importance sampling with a Monte Carlo chain length of 10,000. It was confirmed via the standard error of the -2LL estimates that this Monte Carlo chain was sufficiently long. The corrected Bayesian Information Criterion (BICc) is derived from -2LL with additional terms that penalize for the number of parameters estimated in the model. The model with the smallest BICc value is generally the preferred one.

1.2 Statistical model

Individual parameters were modeled as random variables, with log-normal distributions. The equation for an individual parameter was:

$$\varphi_i = \varphi_{pop} \cdot e^{\eta_i}, \qquad \text{Eq. S1}$$

where φ_{pop} was the population typical parameter and η_i was a normally distributed random variable with mean 0 and standard deviation ω.

The combined error model in Monolix is given by:

$$y_{obs} = y_{pred} + (a + b \cdot y_{pred}) \cdot \varepsilon, \quad \text{Eq. S2}$$

where y_{obs} is the observed variable, y_{pred} the model prediction, and ε is an independent random variable, normally distributed with mean 0 and variance 1. For $cNfL_{CSF}$, the parameter a was set to zero yielding a proportional error model. For brain volume, the parameter b was set to zero yielding an additive error model.

Continuous covariates were modeled with the equation

$$\varphi_i = \varphi_{pop} \cdot \left(\frac{\Omega_{j,t}}{\Omega_{pop}}\right)^{\beta} \cdot e^{\eta_i}, \quad \text{Eq. S3}$$

where $\Omega_{j,t}$ was the covariate of the subject j at time t and Ω_{pop} was the typical population covariate. Categorical covariates were modeled with the equation

$$\varphi_i = \varphi_{pop} \cdot e^{\beta(\Omega_j=\Omega_1)} \cdot e^{\eta_i}, \quad \text{Eq. S4}$$

where $\mathbf{1}_{WJ=WI}$=1 if the individual covariate was in the category and $\mathbf{1}_{WJ=WI}$=0 otherwise. The typical population value for CAG repeats was 42.5 (the mean of the HD cohort).

1.3 Covariate model development

For covariate model building, selected covariates were plotted for visual exploration and the summary statistics was computed. A stepwise forward inclusion and stepwise backward elimination approach was used to determine significant covariates. In the stepwise forward inclusion step, each candidate covariate was included into the model individually and tested for significance. Covariates were considered significant when the change in the -2LL relative to the base model was larger than 3.84, which corresponded to an acceptance level of p=0.05 (chi-squared test on the log-likelihood ratio with 1 degree of freedom). No backwards elimination step was executed as all covariates estimated as significant remained significant upon addition to the base model, as determined with the Wald test at a significance level of p=0.01 and the -2LL.

1.4 Simulations

Simulations were performed in R using the Simulix API (Simulix 2023R1). The simulations included covariate effects, between individual variability, but no observational error. Covariates for each simulated patient were sampled from a random distribution or were fixed to a specific value to investigate the influence of the covariate on the outcome. For each simulation scenario (e.g., subject group with a given number of CAG repeats) 500 individuals were simulated, and summary statistics were calculated to derive the different percentiles of the values across the population.

1.5 Final parameter estimates

Table S1 gives the final parameter estimates of the model.

Description	Parameter (Units)	Estimate (%CV)	RSE (%)
**Fixed effects (population parameters)**			
Intercept of brain volume polynomial $V_{Brain}(t)$; Eq. 4	k0 (mL)	1190 (4)	1.3
1st-order term of $V_{Brain}(t)$; Eq. 4	k1 (mL/yr)	4.84 (9)	6
2nd-order term of $V_{Brain}(t)$ in Healthy Controls; Eq. 4	k2_HC (mL/yr/yr)	0.0902 (8)	6.8
2nd-order term of $V_{Brain}(t)$ in Huntington's Subjects; Eq. 6	k2_HD (mL/yr/yr)	0.125 (6)	2
β coefficient for continuous covariate CAG on k2_HD; Eq. 6	coeffCAG (-)	2.74 (no IIV)	23
Concentration of NfL in brain ($cNfL_{Brain}$); Eq. 3	cNfL_Brain (ug/g)	15.5 (no IIV)	8.1
NfL Clearance rate in CSF in Healthy Controls; Eq. 3	Cl_NfL (uL/min)	303 (30)	fixed
**Standard deviations**			
Intercept of brain volume polynomial $V_{Brain}(t)$; Eq. S1	ω_k0	0.0404	14
Intercept of brain volume polynomial $V_{Brain}(t)$; Eq. S1	ω_k1	0.0897	55
1st-order term of $V_{Brain}(t)$ in healthy controls; Eq. S1	ω_k2_HC	0.0791	44

2nd-order term of $V_{Brain}(t)$ in Huntington's disease subjects; Eq. S1	ω_k2_HD	0.0611	30
NfL Clearance rate in CSF in Healthy Controls; Eq. S1	ω_Cl_NfL	0.297	13
**Covariate coefficients**			
GROUP_manifest on Cl_NfL; Eq. S4	β_Cl_NfL_GROUP_manifest	-1.03	fixed
GROUP_premanifest on Cl_NfL; Eq. S4	β_Cl_NfL_GROUP_premanifest	-0.801	fixed
**Observational error**			
Proportional error NfL concentrations in CSF; Eq. S2	b_cNfL_CSF	0.216	8.2
Constant error whole brain volume; Eq. S2	a_V_Brain (mL)	11	11
Stochastic approx. used for estimation of SE			
%CV from Monolix			
IIV denotes inter-individual variability			

Table S1: Final parameter estimates. See Equations 1-6 of main text and S1-S4 (above) for definitions of the parameters.

The parameter values referring to fixed effects and observational error are presented and discussed in the main text. Graphical representations of the additive and proportional models used to describe the observational error in the brain volume and $cNfL_{CSF}$ data, respectively, are presented in section 2 (Figures S3A and S4A).

1.6 Comparison of linear and quadratic functions for representing the age/time dependence of brain volume.

On an empirical basis we have used a quadratic function to represent the age/time dependence of brain volume in all three subject groups. To assess whether a linear function would have been sufficient to represent these data, we have re-analyzed the brain volume and $cNfL_{CSF}$ data by setting the k2 parameter of Equation 4 to zero. In doing so we allowed the model to estimate covariates for the k0 and k1 parameters to allow for different linear relationships among the three subject groups.

The resulting linear function model showed that all three groups had the same value of k0 (1276 mL) and that the manifest group had a different value of k1 (-4.017 mL/yr) from the healthy controls and pre-manifest groups, which were the same (-1.734 mL/yr). Figures S1 and S2 superimpose the linear and quadratic (polynomial) models on the brain volume and $cNfL_{CSF}$ data, respectively.

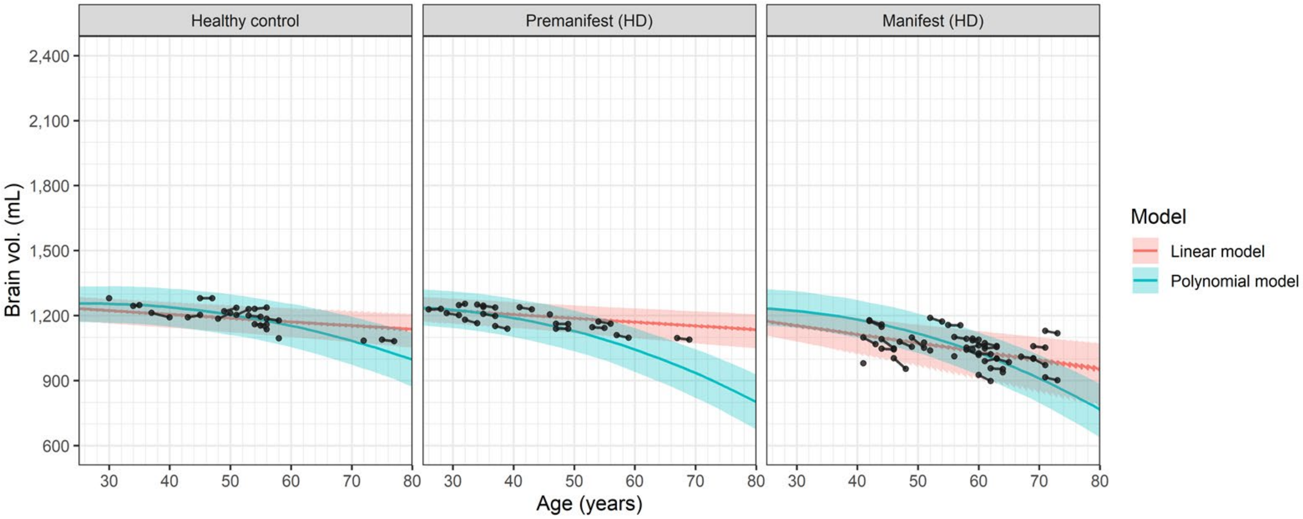

Figure S1. Comparison of a linear function model and quadratic (polynomial model) in representing the age/time dependence of the brain volume data in healthy controls, premanifest and manifest HD subjects. Filled circles represent individual observations with line segments connecting the baseline and 24-month follow up measurements. A small number of data points had no follow up measurement. Shaded areas represent the 90% prediction intervals of the models with the median values shown as lines or curves.

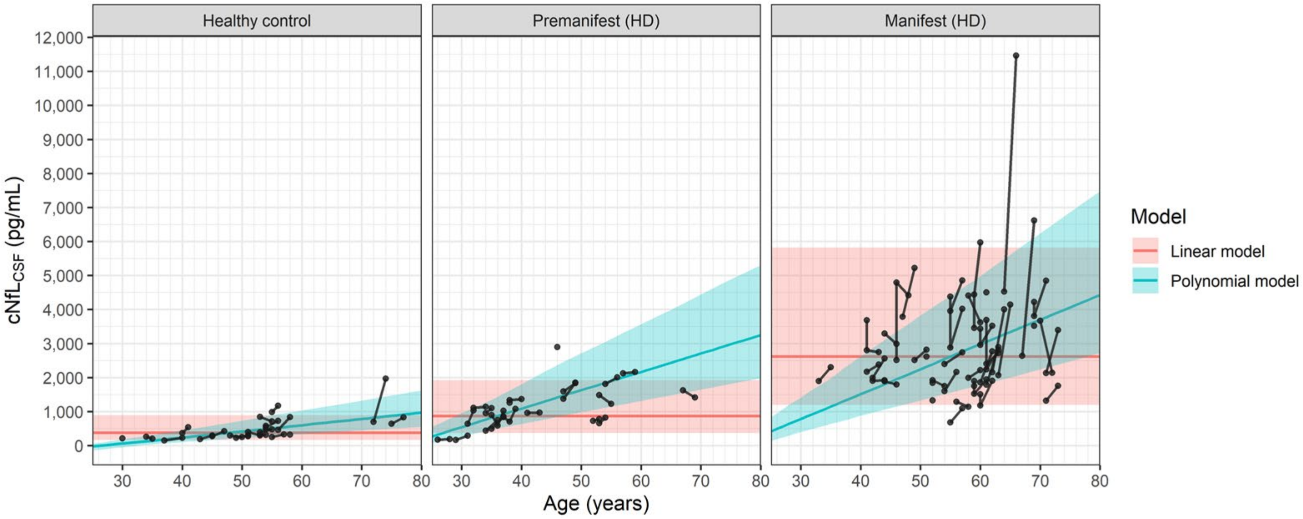

Figure S2. Comparison of a linear function model and quadratic (polynomial model) in representing the age/time dependence of the $cNfL_{CSF}$ data in healthy controls, premanifest and manifest HD subjects. Filled circles represent individual observations with line segments connecting the baseline and 24-month follow up measurements. A small number of data points had no follow up measurement. Shaded areas represent the 90% prediction intervals of the models with the median values shown as dotted lines or curves.

For the brain volume data, the difference between the linear and quadratic (polynomial) models is subtle. However, for the $cNFL_{CSF}$ data the difference between the two models is striking. The flat prediction intervals of the linear model are clearly incompatible with the increasing trends of the quadratic model. The superiority of the quadratic model was confirmed by the BICc parameter, which was 13.6 points lower (3742.65) than the linear model (3756.29), despite the penalty imposed by the larger number of estimated parameters.

2 Supplementary File 2: Graphical assessments of goodness of fit and observational error

The goodness of fit of non-linear mixed effect models such as ours can be assessed graphically in a number of ways. Figures S3A and S4A illustrate observations vs. individual subject predictions for brain volume and NfL concentrations in CSF ($cNfL_{CSF}$), respectively, and the corresponding 90% prediction intervals. Figures S3B and S4B display the associated distributions of the observational error in the data derived from the error models assumed for each variable.

A B

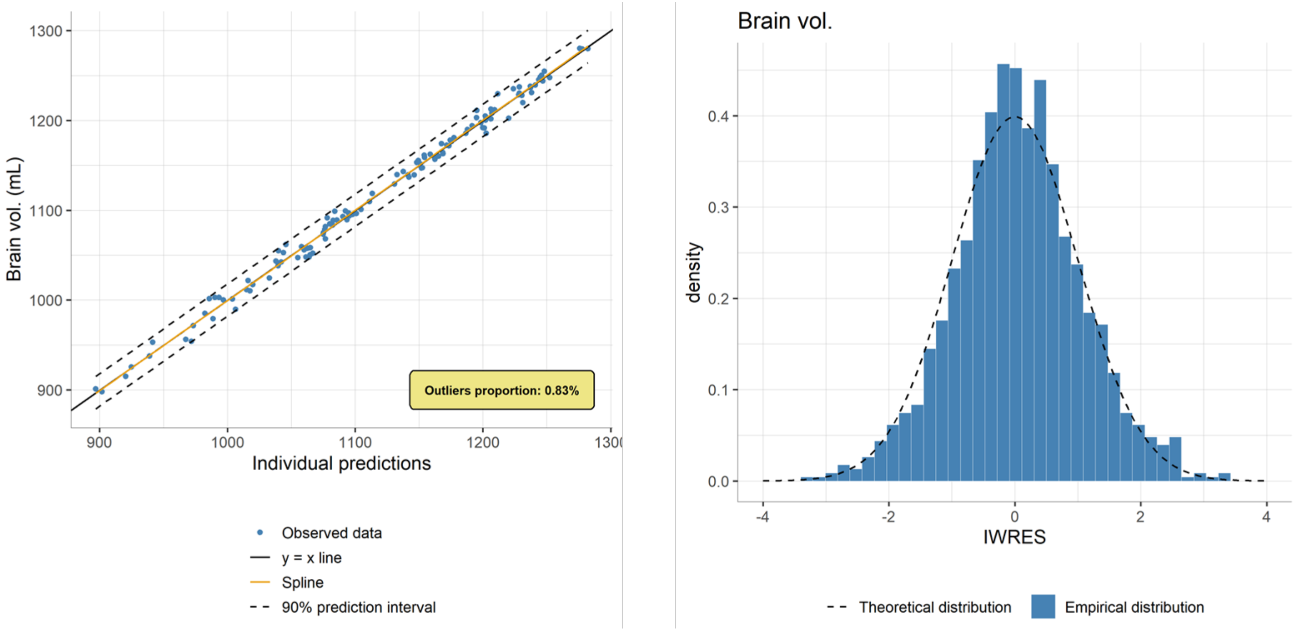

Figure S3. Panel (A) denotes observations of brain volume vs. the individual model predictions derived for each subject. The line of identity is shown in black. A spline function of the data is shown in gold. The 90% prediction interval corresponds to the parallel dashed lines resulting from an additive error model. The proportion of outliers from the 90% prediction interval is 0.83%. Panel (B) shows a histogram of the empirical distribution of the individual weighted residuals (IWRES) compared to the expected theoretical distribution.

A B

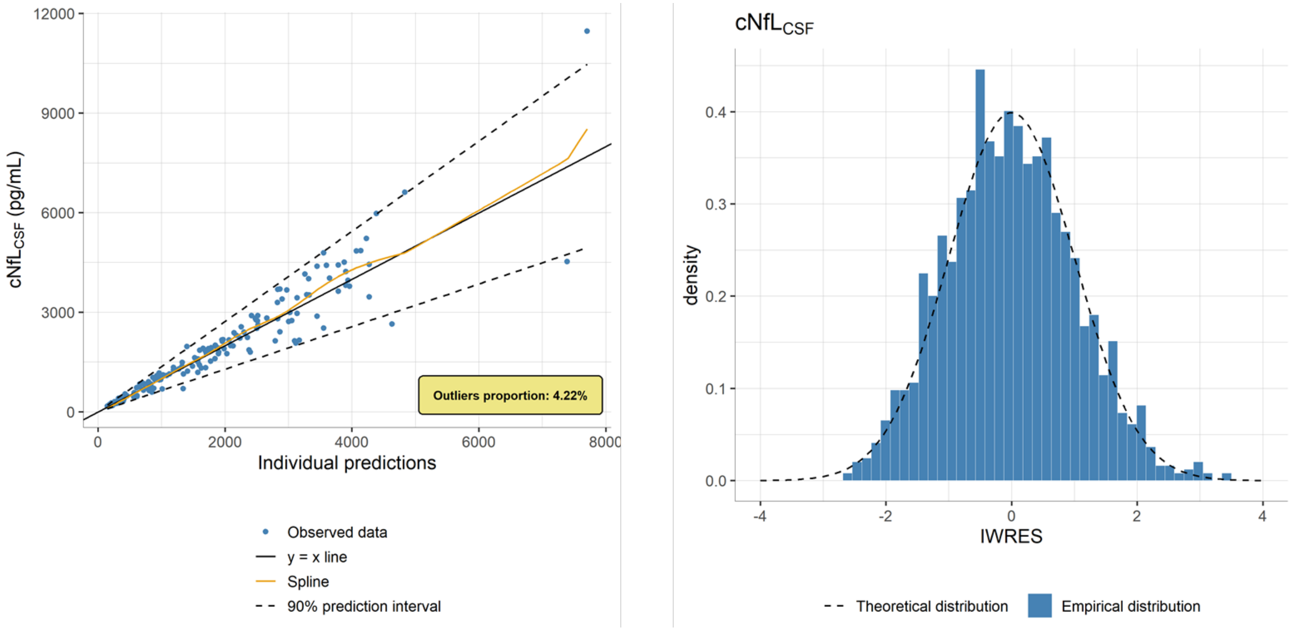

Figure S4. Panel (A) denotes observations of $cNfL_{CSF}$ vs. the individual model predictions derived for each subject. The line of identity is shown in black. A spline function of the data is shown in gold. The 90% prediction interval corresponds to the splayed dashed lines resulting from a proportional error model. The proportion of outliers from the 90% prediction interval is 4.22%. Panel (B) shows a histogram of the empirical distribution of the individual weighted residuals (IWRES) compared to the expected theoretical distribution.

The closeness of the spline functions (gold curves) to the lines of identity in figures S3A and S4A are indicative of an acceptable model fit to each variable. The appropriateness of the additive error model assumed for brain volume and the proportional error model assumed for $cNfL_{CSF}$ is indicated by the small percentages of outliers from the 90% prediction intervals seen in figures S3A (0.83% fall outside the parallel dashed lines) and S4A (4.22% fall outside the splayed dashed lines). In addition, the appropriateness of the error models is further illustrated in figures S3B and S4B where the empirical distributions of the individually weighted residuals (IWRES) correspond well to the theoretical (Normal) distributions.

Additional graphical assessments of the goodness of fit are based on the visual predictive checks (VPCs) generated by the Monolix API (Monolix 2023R1).

Figures S5 and S6 illustrate VPCs for the brain volumes and NfL concentrations in CSF, respectively. In each case, panels (A), (B) and (C) correspond to data for the healthy controls, premanifest HD subjects and manifest HD subjects respectively.

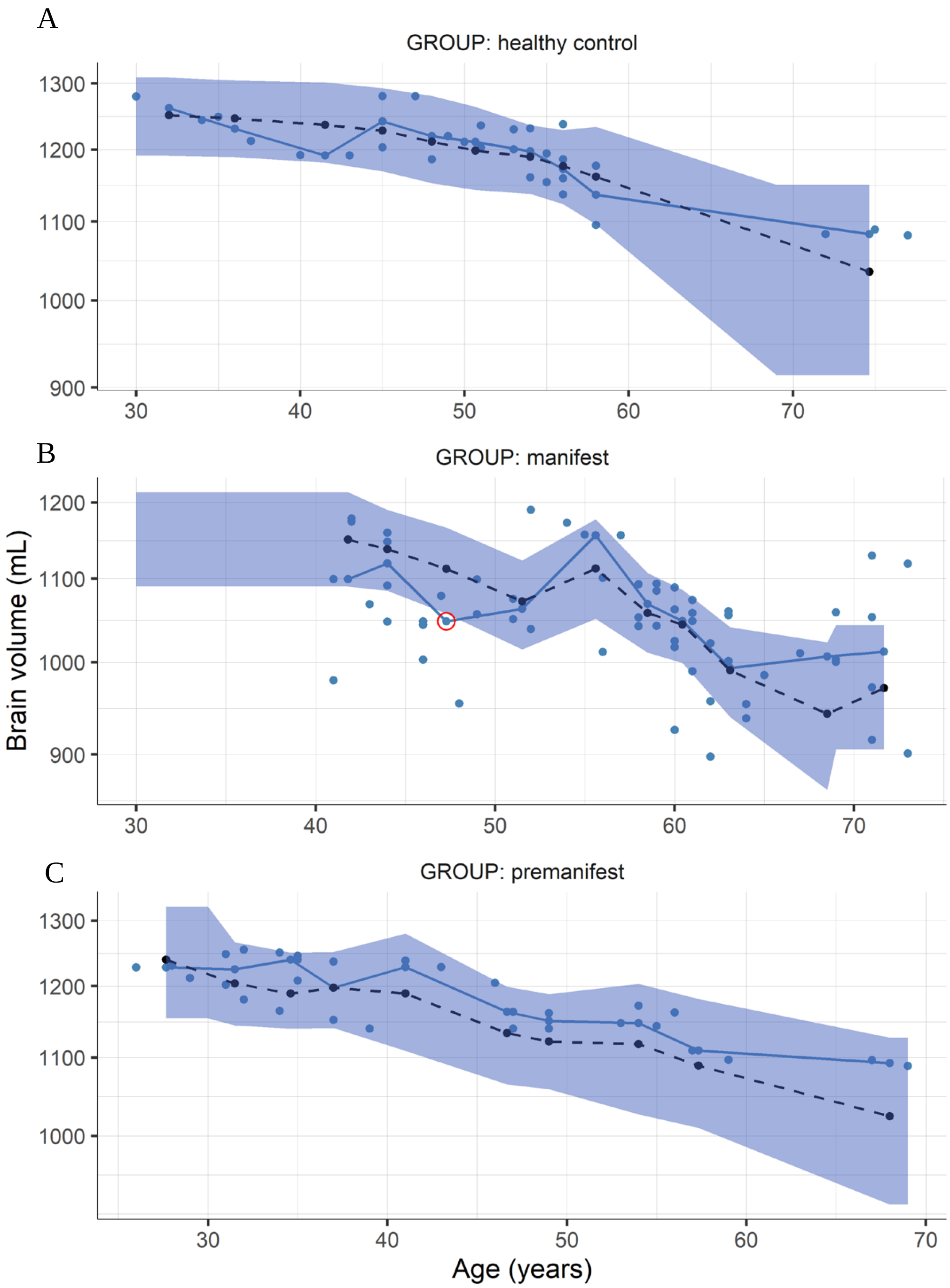

Figure S5. VPCs for brain volume. Panel (A) shows group of healthy controls. Panel (B) shows group of manifest HD subjects. Panel (C) shows manifest HD subjects. Blue-filled circles represent data from individual subjects. Blue line segments are the median values of the data. Dashed line segments are the 50th percentile of the model simulations. Blue regions cover the 10th to the 90th percentile of the simulations. Median values circled in red are considered outliers.

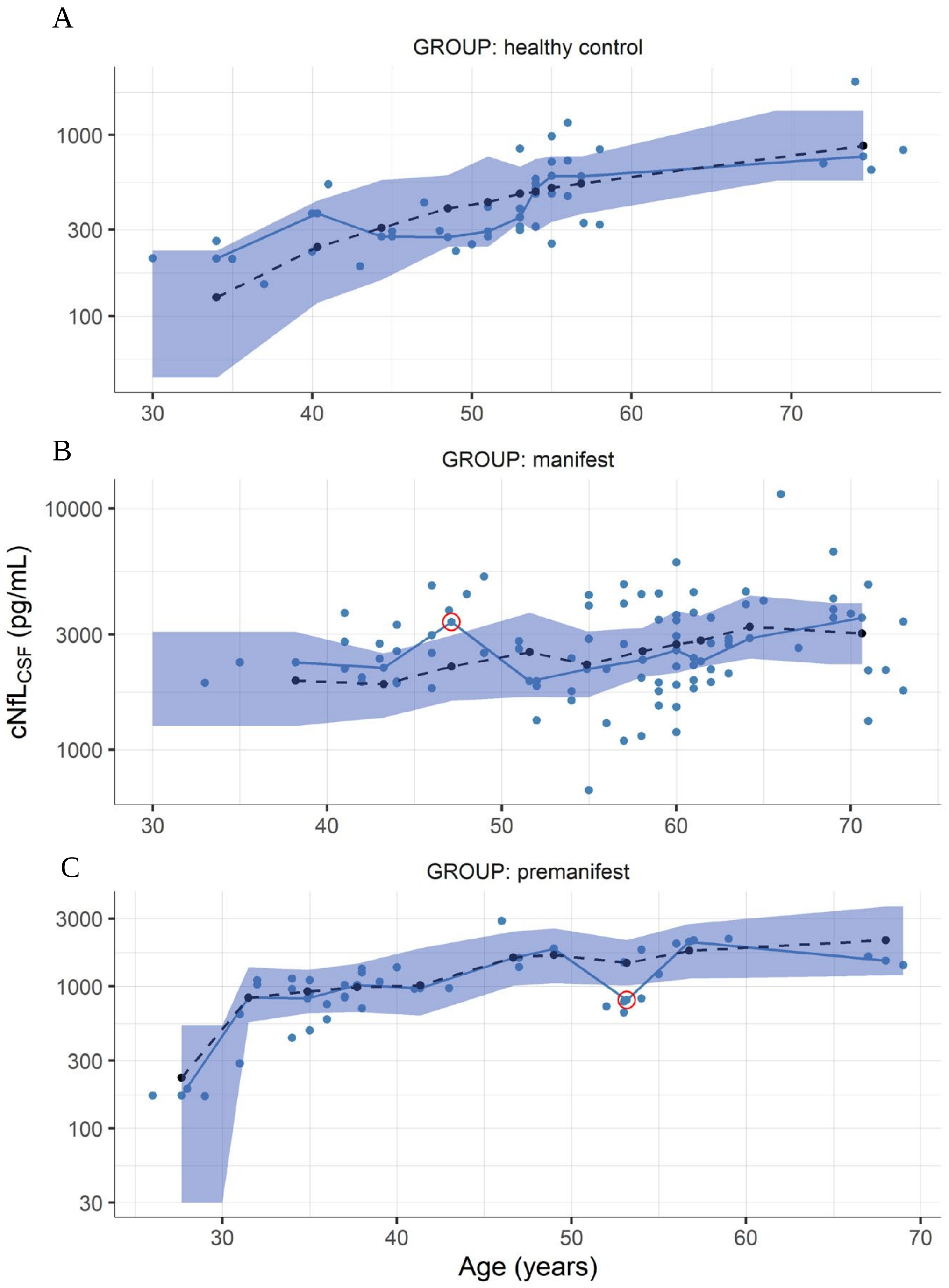

Figure S6. VPCs for NfL concentration in CSF (cNfL$_{CSF}$). Panel (A) shows group of healthy controls. Panel (B) shows group of manifest HD subjects. Panel (C) shows manifest HD subjects. Blue-filled circles represent data from individual subjects. Blue line segments are the median values of the data. Dashed line segments are the 50th percentile of the model simulations. Blue regions cover the 10th to the 90th percentile of the simulations. Median values circled in red are considered outliers.

With the exception of a small number of outliers, the VPCs for brain volume and $cNfL_{CSF}$ show good correspondence between the data and model predictions in all three subject groups; attesting to the goodness of fit to the data.

3 Supplementary File 3: Effect of CAG repeat length on brain volume and NfL concentrations in CSF

As further illustrations of the consistency between the model predictions and the data, Figures S7 and S8 show the influence of CAG repeat length on the age-dependence of brain volume and NfL concentrations in CSF ($cNfL_{CSF}$), respectively, in premanifest and manifest HD subjects.

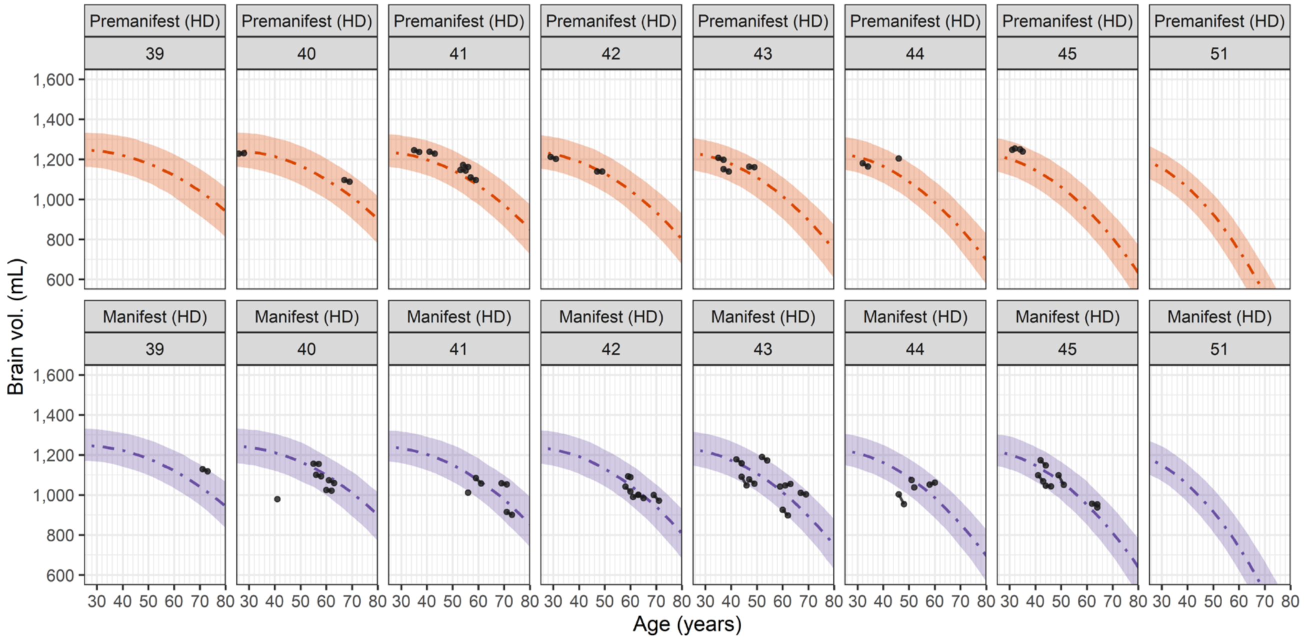

Figure S7. Effect of CAG repeat length on the age-dependence of brain volume. Upper panels correspond to premanifest HD subjects grouped according to their CAG repeat lengths. Lower panels correspond to manifest HD subjects grouped in the same way. Individual data points are shown as black circles, connected by line segments for longitudinal observations. Orange and purple dashed curves are the predicted median trajectories of the model for each CAG value. Shaded regions correspond to the 90% prediction intervals of the model.

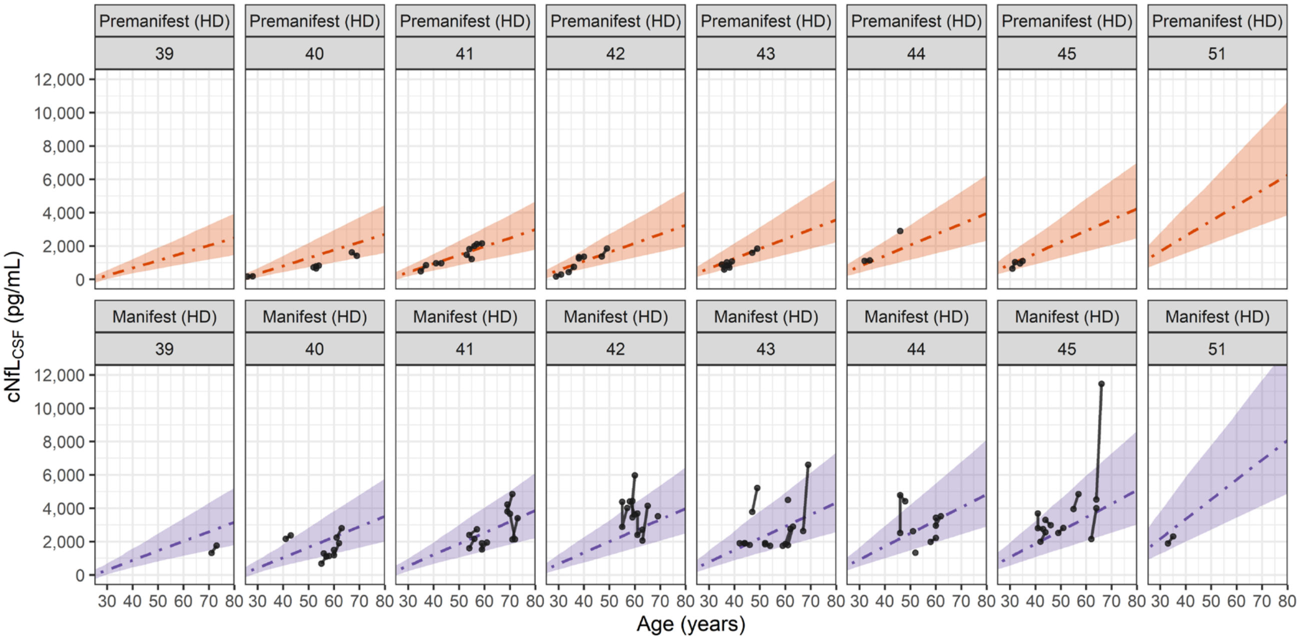

Figure S8. Effect of CAG repeat length on the age-dependence of NfL concentrations in CSF ($cNfL_{CSF}$). Upper panels correspond to premanifest HD subjects grouped according to their CAG repeat lengths. Lower panels correspond to manifest HD subjects grouped in the same way. Individual data points are shown as black circles, connected by line segments for longitudinal observations. Orange and purple dashed curves are the predicted median trajectories of the model for each CAG value. Shaded regions correspond to the 90% prediction intervals of the model.

The increasing downward curvature of the brain volume trajectories and increasing slope of the $cNfL_{CSF}$ trajectories both result from the strong dependence of the k2_HD parameter on CAG given in Equation 6 of the main text.

4 Supplementary File 4: Modeling the Svennerholm brain weight study

Additional support for the 2nd order polynomial used to described the dependence of brain volume on age (or equivalently time) is shown here by modeling data from Svennerholm's study (Svennerholm 1997) of brain weight in men and women ranging in age from 20 to 100 years, with no prior history of neurological or psychiatric impairment (or post-mortem evidence of neuropathology). Converting Svennerholm's data to brain volume (using a density of 1.03 g/mL of tissue) and limiting the age range from 26 to 77 years (as in the HD-CSF study), we show in Figure S9 a comparison of the Svennerholm data with the predicted median and 90% confidence interval from our model. It is important to note that the only parameter re-estimated for this prediction was k_0, the intercept of the brain volume polynomial, which had a value of 1501 mL (compared to the previous estimate of 1190 mL). The values of k_1 and k_2, which describe the rate of neurodegeneration and its acceleration with age, are the same as the previous values given in Table S1.

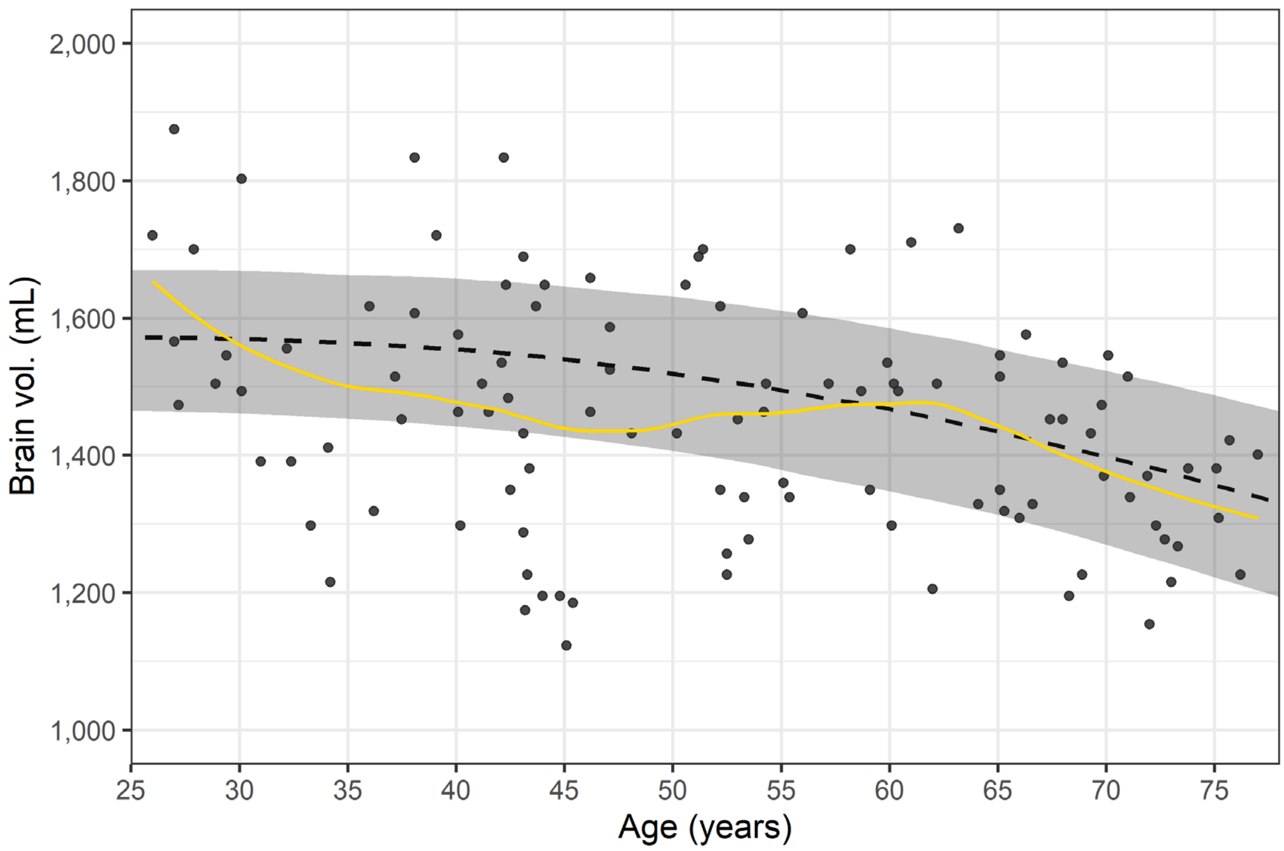

Figure S9. Comparison of brain volume data derived from the Svennerholm study for men and women ranging in age from 26 to 77 years with model predictions based on the 2nd order polynomial (Equation 4 of the main text). Dashed curve is the predicted median and shaded area is the 90% confidence interval. Yellow curve is spline function describing the age-dependence in the data.

The close correspondence of the spline function to the model trajectory is strongly supportive of our prior estimates of k_1 and k_2. The larger value of k_0 estimated from the Svennerholm data is most likely a result of the post-mortem gravimetric methodology used by Svennerholm and the fact that the raw data were not adjusted for total intracranial volume as was done in the HC-CSF study.

Reference:

Svennerholm, L., Boström, K. and Jungbjer, B., 1997. Changes in weight and compositions of major membrane components of human brain during the span of adult human life of Swedes. Acta neuropathologica, 94, pp.345-352.
